# Selenoprotein P expression in glioblastoma as a regulator of ferroptosis sensitivity: preservation of GPX4 via the cycling-selenium storage

**DOI:** 10.1038/s41598-024-51259-5

**Published:** 2024-01-05

**Authors:** Xi Zheng, Takashi Toyama, Stephanie Siu, Takayuki Kaneko, Hikari Sugiura, Shota Yamashita, Yoshiteru Shimoda, Masayuki Kanamori, Kotoko Arisawa, Hidenori Endo, Yoshiro Saito

**Affiliations:** 1 Laboratory of Molecular Biology and Metabolism, Graduate School of Pharmaceutical Sciences, Tohoku University, 6-3 Aoba, Aramaki, Aoba-ku, Sendai, 980-8578 Japan; 2 Department of Neurosurgery, Graduate School of Medicine, Tohoku University, 2-1 Seiryo Aoba-ku, Sendai, 980-0872 Japan

**Keywords:** Cell biology, Health care

## Abstract

Glioblastoma (GBM) is one of the most aggressive and deadly brain tumors; however, its current therapeutic strategies are limited. Selenoprotein P (SeP; SELENOP, encoded by the *SELENOP* gene) is a unique selenium-containing protein that exhibits high expression levels in astroglia. SeP is thought to be associated with ferroptosis sensitivity through the induction of glutathione peroxidase 4 (GPX4) via selenium supplementation. In this study, to elucidate the role of SeP in GBM, we analyzed its expression in GBM patients and found that SeP expression levels were significantly higher when compared to healthy subjects. Knock down of SeP in cultured GBM cells resulted in a decrease in GPX1 and GPX4 protein levels. Under the same conditions, cell death caused by RSL3, a ferroptosis inducer, was enhanced, however this enhancement was canceled by supplementation of selenite. These results indicate that SeP expression contributes to preserving GPX and selenium levels in an autocrine/paracrine manner, i.e., SeP regulates a dynamic cycling-selenium storage system in GBM. We also confirmed the role of SeP expression in ferroptosis sensitivity using patient-derived primary GBM cells. These findings indicate that expression of SeP in GBM can be a significant therapeutic target to overcome anticancer drug resistance.

## Introduction

Glioblastoma (GBM) accounts for 82% of malignant glioma cases and is one of the most aggressive and fast-growing brain tumors^1^. GBM can lead to neurologic symptoms including weakness, visual and sensory changes, mood changes, memory or executive function, language disorder, and headaches, among others^2^. The current standard of care for glioma is surgical resection followed by radiotherapy and temozolomide chemotherapy^3^, however median survival rate after treatment is only around14.6 months^3,4^. Thus, there is an urgent need to develop a new therapeutic strategy for GBM. Recently, ferroptosis, an iron-dependent oxidative stress-mediated form of cell death, received much attention in the cancer research field^5,6^, and interestingly it was reported that ferroptosis is also the underlying mechanism of action of temozolomide (TMZ)^7^. TMZ is DNA-alkylating (methylating) agent and it susceptibility is regulated by O6-methylguanine methyltransferase (MGMT) expression, which plays repairer of DNA methylation^8^. Recent studies suggested that many novel independent mechanisms play important role in TMZ resistance, e.g., autophagy^3,9^, however its contribution to ferroptosis-resistance is little known.

Ferroptosis was first described in 2012, and is characterized by iron-dependent lipid peroxidation and the generation of free radical-mediated lipid peroxidation products that occur directly before cell disintegration and cell death^10,11^. Since reduction of the lipid peroxidation process is related to chemotherapy sensitivity, inhibition of this process would be beneficial to overcome treatment resistance^12^. Glutathione peroxidase 4 (GPX4), a selenium-containing enzyme^13^, plays reduction of phospholipid hydroperoxides. The regulatory mechanism of GPX4 expression in cancer cells has been studied; however, the underlying mechanism is complicated due to the unique regulation of its translation system, and thus it remains poorly elucidated.

Selenium is an essential trace element involved in many physiological processes, such as energy metabolism, immune function, antioxidant defense, and neuronal cell survival^14,15^. The incorporated selenium is metabolized to selenocysteine through a de novo metabolic pathway, in which proteins such as selenocysteine lyase, SEPHS2, PSTK, SPS2, and SEPSECS are involved^16,17^. The selenocysteine residue synthesized on tRNASec is translated in the presence of a stable loop structure called the selenocysteine-insertion sequence (SECIS) in the 3' untranslated region of selenoprotein mRNA^18^. The human genome encodes 25 selenoproteins, including five types of GPX and three types of thioredoxin reductase (TXNRD). Both GPX4 and TXNRD1 are reported to contribute to cancer proliferation and resistance to ferroptosis^19^. The regulatory mechanism of selenoproteins at the protein level is known to be highly dependent on selenium supply, as well as on mRNA levels because reduction of selenocysteine-tRNASec causes the codon corresponding to selenocysteine (termination codon UGA) to be recognized as a termination codon, resulting in mRNA degradation, translation arrest, and read-through^19,20^. Therefore, more attention should be given to whether comprehensive and large-scale analyses such as RNA-sequencing precisely reflect the variation of selenoproteins at the protein level.

Our research group previously reported that treatment with selenoprotein P (SeP; SELENOP, encoded by the *SELENOP* gene) siRNA resulted in a ferroptosis-like cell death in insulinoma MIN6 cells^21^, and a recent study also indicated that neuroblastoma, a neuronal tumor, requires SeP as a selenium source to confer resistance against ferroptosis through GPX4 expression^22^. SeP is a selenium-containing extracellular protein that is mainly secreted from hepatocytes into plasma, and incorporated into peripheral tissues^23^. In the central nervous system, SeP expression is observed in several cell types, including neurons and ependymal cells^24^. Notably, the expression of SeP is relatively high in astrocytes^25^, however the physiological/pathophysiological role of SeP expression in astrocytes and the central nervous system is not fully elucidated. SeP is a unique selenoprotein that contains 10 Sec residues per polypeptide, whereas other selenoproteins usually have only one^26^. This feature endows SeP with the distinct function of delivering Se to cells/tissues. Whole-body SeP knockout causes a reduction of selenium levels in the brain^27^, while conditional gene knockout of SeP in the liver does not^28^, suggesting that SeP production in the central nervous system plays a role in brain selenium retention. SeP is recognized by receptors such as LRP8 (ApoER2), incorporated into cells and degraded by lysosomes to produce selenocysteine^29,30^. SeP synthesized in the brain is incorporated into other brain cells and used to synthesize selenoproteins, which help maintain Se concentrations in the brain^25,31^. This system is called the SeP cycle, which is a cycling-selenium storage system used to retain selenoprotein levels in cells. The similar phenotype between SELENOP and ApoER2 KO mice implies that ApoER2 is a significant mediator of the SeP cycle^29,32^. Recently, a preferential selenium transport pathway involving SeP and high-affinity ApoER2 in a Sec lyase–independent manner has been discovered^33^. While SeP is crucial for selenium metabolism, excess SeP production is associated with several diseases such as type 2 diabetes, dementia, and pulmonary hypertension^34^. Recent evidence suggests the implication of excess SeP in cancer^35,36^; however, its understanding is limited.

In the present study, we found that SeP regulates GPX4 expression in GBM, and contributes to ferroptosis resistance through the formation of a cycling-selenium storage system. This study not only reveals a unique selenium utilization and metabolism pathway, but also defines SeP as a promising therapeutic target for GBM treatment to enhance sensitivity against anti-cancer drugs.

## Results

### Expression of *SELENOP* in tissues of GBM patients

**Figure 1:**
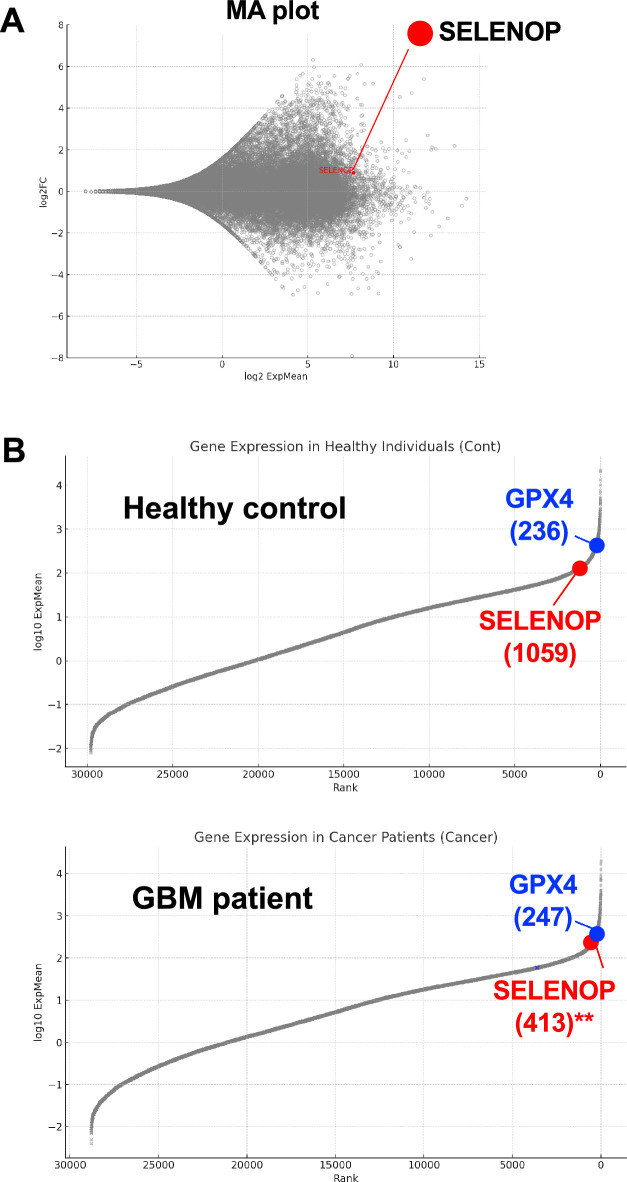
*SELENOP* expression in GBM patients. MA plot of the genes in GBM patients compared with healthy control (**A**). *SELENOP* gene is indicated in red. Expression gene rank of healthy control and GBM patients are plotted (**B**). Gene rank of *GPX4* (indicated as blue) and *SELENOP* (indicated as red) are listed under each gene caption.

We analyzed RNA-Seq data of GBM patients and found that *SELENOP* expression was relatively higher in tissues of GBM patients compared to healthy subjects (Fig. 1A). Gene ranking alignment suggested that expression rank of *SELENOP* is significantly higher in GBM patients (rank = 413), while *GPX4* mRNA level remained unchanged (Fig. 1B). The data from this comprehensive approach suggests that *SELENOP* levels increase at the mRNA level in GBM patients.

### SeP expression contributes to ferroptosis resistance in cultured GBM

**Figure 2:**
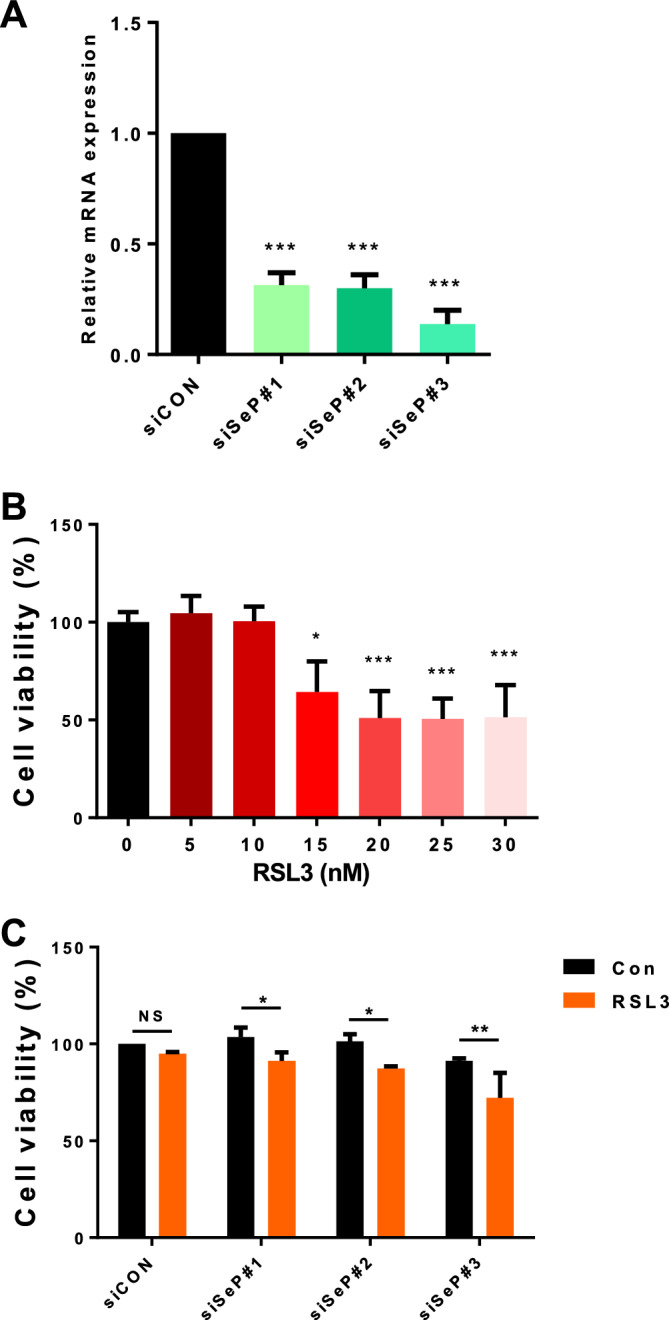
Sep inhibits cell death induced by RSL3, a ferroptosis inducer, in T98G. T98G cells were transfected with three different SeP siRNAs and 48 h post-treatment, SeP mRNA levels were measured by RT-qPCR (**A**). T98G cells were treated with control siRNA (siCON), and RSL3 was added at the indicated concentrations. After 24 h, cell viability was measured (**B**) (data were shown as mean ± S.D.; n = 3). Cells were pre-treated with three different siRNAs against SeP for 24 h, then treated with 10 nM RSL3 for an additional 24 h, and cell viability was measured (**C**). Data are presented as the mean ± S.D. Statistical significance was assessed by Dunnett’s test (**A**,**B**) and t-test (**C**). *p < 0.05, **p < 0.01, ***p < 0.001 vs. control.

In several types of cells, SeP expression plays a crucial role in cellular selenium metabolism and antioxidant defense via the preservation of GPXs ^26,36^. We investigated the role of SeP expression in ferroptosis sensitivity in the glioblastoma cell line T98G. Knockdown efficiency of SeP was confirmed by qPCR (Fig. 2A), and the concentration of RSL3, an anti-cancer drug known to induce ferroptosis, was examined in negative control siRNA treated cells (Fig. 2B). From these results, we set the maximal RSL3 concentration that did not affect cell viability as 10 nM. Next, we examined cell viability following RSL3 treatment, and found that the effects of RSL3 were enhanced by SeP knockdown (Fig. 2C). These results indicate that SeP expression may confer protection against ferroptosis in glioblastoma cells.

### SeP expression is responsible for maintaining GPX4 expression at the protein level in cultured GBM

**Figure 3:**
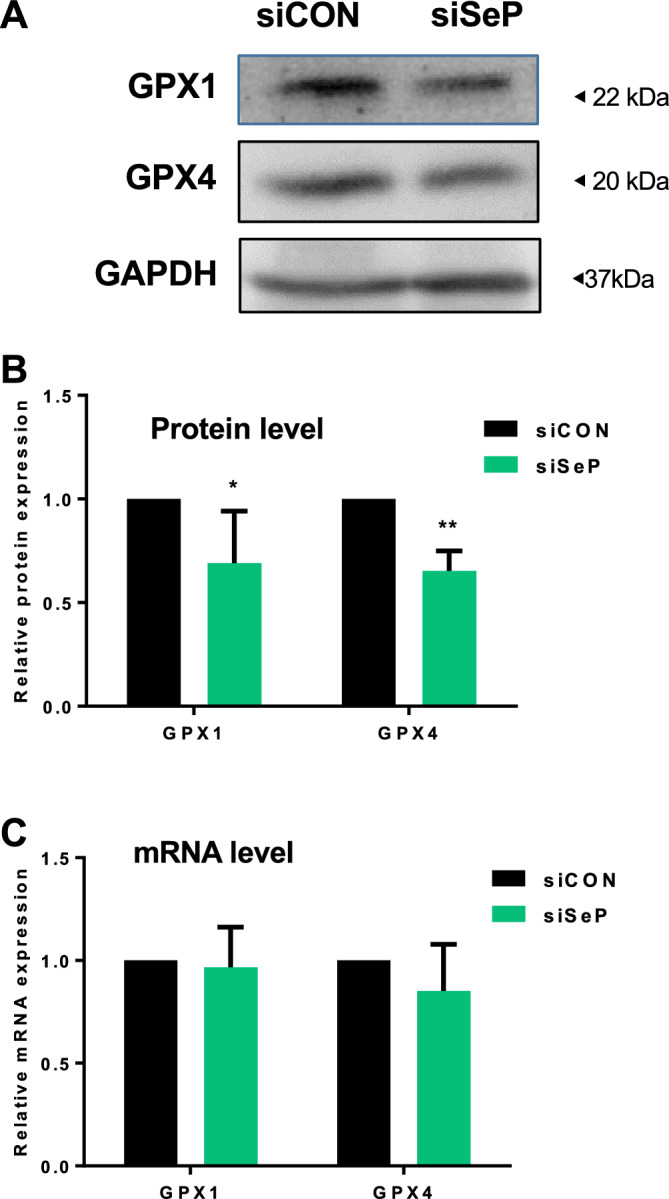
SeP is required for maintaining of glutathione peroxidase expression in T98G. Cells were transfected with SeP siRNA (#3 siRNA was used as the representative) for 48 h. Protein expression of selenoproteins (GPX1 and GPX4) were measured by Western blotting (**A**). Protein content of each selenoprotein was normalized using GAPDH, and relative expression levels are shown with the control as 1 (**B**). Data are shown as mean ± S.D; n = 3. Under the same conditions, mRNA levels of each selenoprotein were measured by RT-qPCR (**C**) (data are shown as mean ± S.D; n = 3). Statistical significance was assessed by the *t*-test. *p < 0.05, **p < 0.01 vs. control.

It has been reported that the ferroptosis regulatory system (GPX4) and anti-oxidative enzymes (GPX1) contribute to anti-cancer drug resistance^37,38^. We hypothesized that SeP expression in GBM would upregulate anti-oxidative selenoproteins and thereby promote ferroptosis resistance. To address this issue, we examined protein and mRNA levels of GPX4 and GPX1 in SeP siRNA-treated cells. The results shown in Fig. 2 indicate that although the three different siRNAs against SeP showed the same phenotype, siRNA #3 was the most efficient and thus was used as the representative siRNA in the following experiments. The results indicated that protein levels of GPX1 and GPX4 decreased significantly following SeP knockdown (Fig. 3A and B), while mRNA levels did not change (Fig. 3C). This suggests that SeP expression is involved in maintaining GPX protein levels, at least in cultured cells.

### Selenium supplementation increases ferroptosis resistance in SeP siRNA-treated GBM

It has been suggested that selenium supply increases selenoprotein levels at both transcriptional and translational levels. Therefore, we hypothesized that suppression of SeP expression in GBM would induce a decrease in GPX expression and increase susceptibility to ferroptosis via selenium depletion, and examined whether the above could be restored by the addition of selenite as a selenium donor.

**Figure 4:**
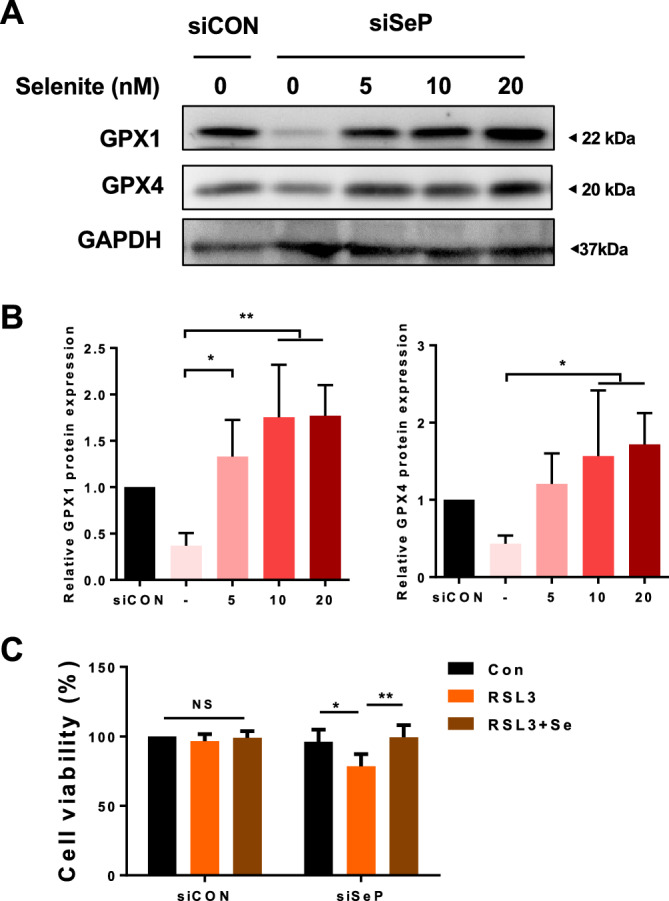
Selenium supplementation rescues sensitivity against RSL3 in SeP siRNA transfected cells. T98G cells were transfected with siRNA for 24 h and incubated with the indicated concentration of selenite for an additional 24 h. The protein was extracted, and GPX levels were detected by Western blot (**A**). Protein content of each selenoprotein was normalized using GAPDH, and relative expression levels are shown with the control as 1 (**B**). After SeP was knocked down, RSL3 or selenite 10 nM was added and incubated for 24 h before cell viability was measured (**C**). Data are presented as the mean ± S.D; n = 3. Statistical significance was assessed by Dunnett’s test (**B**) Tukey’s HSD (**C**). *p < 0.05, **p < 0.01 vs. control.

Protein expression of GPX, which was reduced by the inhibition of SeP, was restored to the same level as the control after treatment with 5–20 nM of selenite (Fig. 4A). The increased susceptibility to ferroptosis induced by SeP knockdown was also restored to the same level as the control after addition of 10 nM selenite (Fig. 4B). These results suggest that basal SeP expression contributes to the maintenance of GPX protein expression and promotes ferroptosis resistance through the supply and retention of selenium.

### Effect of SeP siRNA on ferroptosis resistance in patient-derived primary GBM

**Figure 5:**
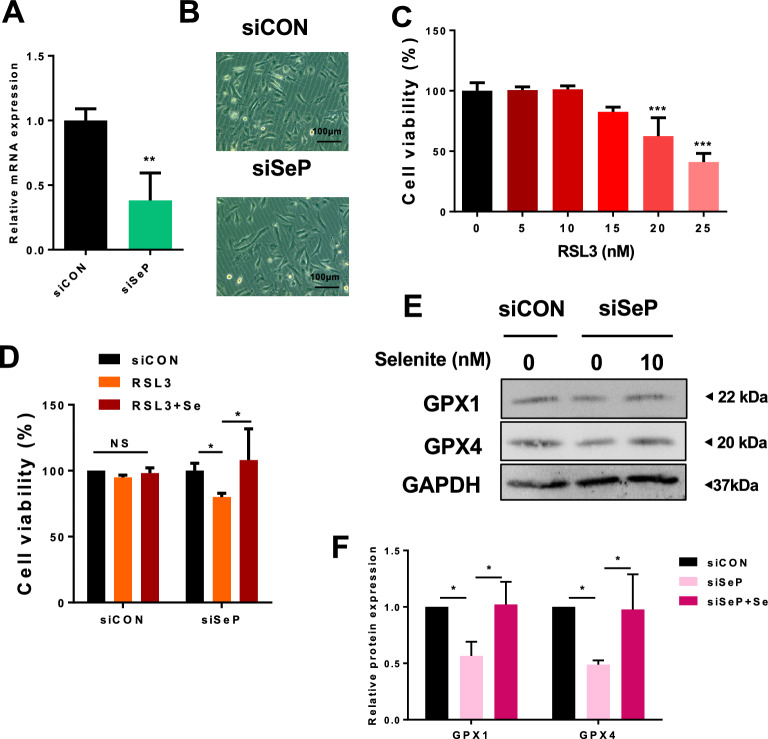
Patient-derived GBM express SeP and its inhibition enhances RSL3-induced cell death. Patient-derived GBM cells were transfected with SeP siRNA for 48 h, and SeP mRNA levels were determined by RT-qPCR (A), and cellular morphology were shown in (**B**). The cells were treated with the indicated concentration of RSL3 for 24 h, and cell viability was determined by alamarBlue assay (**C**). Cells were transfected with siRNAs against SeP for 24 h, then treated with 15 nM RSL3 for an additional 24 h, and cell viability was measured (**D**). Cells were transfected with SeP siRNA for 48 h. Protein expression of selenoproteins (GPX1 and GPX4) were measured by Western blotting (**E**). Protein content of each selenoprotein was normalized using GAPDH, and relative expression levels are shown with the control as 1 (**F**). Data are shown as mean ± S.D.; n = 3. Data are presented as the mean ± S.D. Statistical significance was assessed by *t*-test (**A**), Dunnet’s test (**C, F**), Tukey’s HSD (**D**). *p < 0.05, **p < 0.01, ***p < 0.001 vs. control.

To confirm the role of SeP in ferroptosis resistance, we used primary GBM cells cultured from patient-isolated tissue and treated them with SeP siRNA. SeP mRNA levels were markedly reduced by SeP siRNA treatment (Fig. 5A). In the condition, cellular morphology was not affected (Fig. 5B). Ferroptosis sensitivity of the control siRNA-treated cells was also verified, and we found that the cells were sensitive to treatment with > 15 nM RSL3 (Fig. 5C). Thus, we treated control and SeP siRNA-transfected cells with 15 nM RSL3 and examined cell viability. As with T98G, SeP inhibition decreased GPX4 expression (Fig. 5E), which led to a significant increase in ferroptosis sensitivity(Fig. 5C), but addition of 10 nM selenite recovered both GPX levels and cell viability (Fig. 5D and E). These results indicate that SeP plays a crucial role in maintaining GPX expression and ferroptosis resistance in GBM.

## Discussion

**Figure 6:**
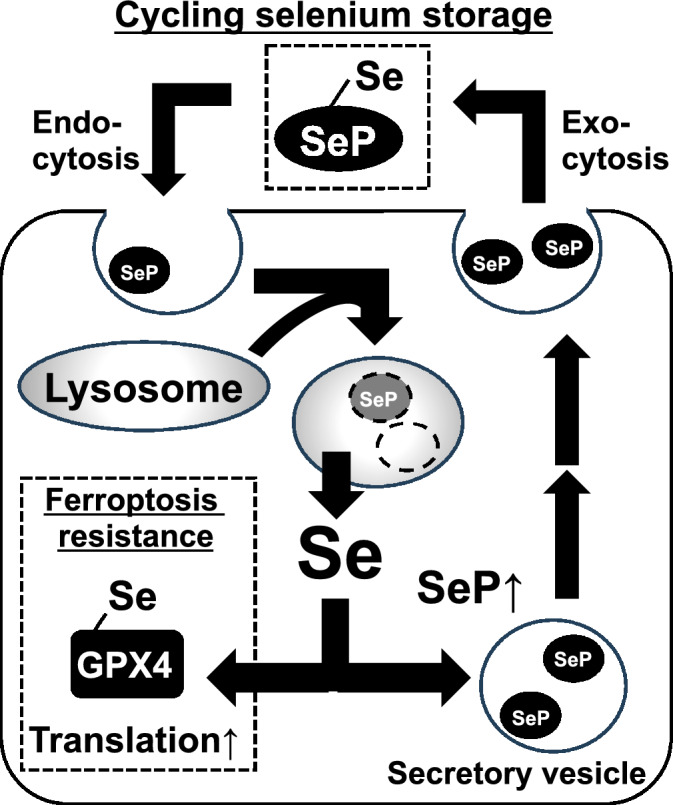
Scheme of SeP-mediated cycling storage of selenium. SeP produced by GBM is involved in maintaining its own GPX expression (Fig. 3). This maintains resistance to ferroptosis (Fig. 2). SeP is degraded by lysosomes to provide intracellular selenium, which is thought to be involved in maintaining GPX expression, but some SeP is also known to be re-released externally by recycling endosomes.

The present study reveals that SeP, which is highly expressed in GBM, contributes to ferroptosis resistance by maintaining GPX expression at the protein level. Our findings identify SeP as a candidate therapeutic target for drug resistant-GBM (Fig. 6).

In GBM prognosis, p53 mutations and the expression of O6-methylguanine DNA methyltransferase (MGMT) gene are important for its treatment. MGMT-promoter methylation (negative regulation of MGMT) and other molecular abnormalities and imaging findings have made it possible to predict prognosis and treatment response in some cases, but accurate evaluation is still difficult. In this study, we validated the expression of SeP in various GBM cell-line which p53 is mutated and MGMT expression is regulated both positively and negatively. The results showed relatively high SeP expression in p53 mutated T98G and U251 (Fig. S1). The highest SeP expression was observed in primary GBM cells with negative p53, suggesting that p53 and SeP may be related in some way. At least, since the suppression of SeP expression did not affect the expression of MDM2 or MDM4, which are p53 regulators (Fig. S2), there is no involvement in this respect, and further detailed verification is needed. MGMT expression of T98G is positive while negative in U251^39,40^. However, even in such cells, suppression of SeP expression reduced GPX expression and increased RSL3 sensitivity as well as T98G cells (Fig. S3A–C). Taken together with the results from primary cells with high SeP expression, the data from at least three cell types suggest that cells with high SeP expression are commonly involved in conferring ferroptosis resistance through selenium retention at least regardless of the expression of MGMT.

Current cancer treatments do not effectively overcome resistance to chemotherapy drugs, and many studies have shown that induction of ferroptosis may be a novel cancer treatment strategy^41^. Temozolomide (TMZ) is currently the main drug used for treatment of GBM, however the prognosis of GBM patients remains poor, mainly due to TMZ resistance^42^. Immunohistochemical analysis of primary and recurrent GBM sample pairs from 24 patients receiving standard adjuvant chemotherapy and radiotherapy showed that recurrent tumors had increased vulnerability to oxidative stress and ferroptosis, and that GBM cells were more susceptible to ferroptosis than microglial cells^43^. In other words, TMZ-resistant cells were more likely to induce ferroptosis^7,44^, and enhancing ferroptosis sensitivity could enhance the toxicity of temozolomide^45^. Thus, ferroptosis is a promising target to improve cancer immunotherapy in GBM. Studies have shown that elevated SeP levels are not only a consequence, but also contributes to the progression of certain diseases^36^. Therefore, if expression of SeP in the brain can be suppressed, drug and treatment resistance of GBM can be overcome. We also examined the contribution of SeP to TMZ toxicity in T98G, but were unable to validate this as no cell death was observed even at up to 400 µM (Fig. S4). Since TMZ tolerance is regulated by MGMT, it would be useful to examine the relationship between MGMT expression and SeP expression, as well as the contribution of MGMT to ferroptosis. ApoER2 (LRP8) is the receptor for SeP in the brain, and the expression of this receptor may have a similar significance. It has been reported that ApoER2 mediated selenocysteine uptake and contributed to the drug-resistance in neuroblastoma^22^. It seems important to address both ligand and receptor side to address the ferroptosis resistance of GBM, which we would like to address in the future.

Nonsense-mediated RNA decay (NMD) is often thought to be the contributing factor to the selenium-dependent regulation of selenoprotein mRNA^46,47^. Selenocysteine is an analog of cysteine, with selenium in place of sulfur, and is encoded by the termination codon UGA^48^. Insertion of selenocysteine into proteins requires a specific cis-acting stem-loop control element called SECIS, which is located in the 3'-untranslated region of the eukaryotic selenoprotein mRNAs, allowing translational read-through of the UGA termination codon^19^. Although the amount of mRNA for many selenoproteins is not dependent on selenium concentration, translation of selenoproteins can be aborted at the UGA codon, resulting in incomplete and non-functional proteins^20^. Since selenoprotein variation in tumors may not be fully detected by approaches such as RNA-Sequence analysis, it is important to evaluate proteomics and protein activity as well.

Numerous studies have reported that GPX4 contributes to ferroptosis resistance via inhibition of lipid peroxidation^49^. On the other hand, although GPX1 has not been reported to be involved in ferroptosis, is thought to contribute to worsening prognosis, tumor growth, and treatment resistance in several cancers, including breast and bladder cancer, via its antioxidative activity^50–52^. Therefore, GPX family members may contribute to the malignant transformation and progression of GBM in a complex manner.

The effect of SeP knockdown on cellular selenium contents should be measured, however, basal selenium levels in cultured T98G cells are difficult to analyze. Regardless, given that the reduced GPX expression in SeP KD was restored by selenite addition, it is at least conceivable that this was not due to reduced selenoprotein synthesis or selenium metabolism by SeP KD, but rather a reduced selenium supply via SeP. SeP is thought to move dynamically in and out of cells following interaction with ApoER2^53^. Therefore, SeP forms a dynamic "cycling-selenium storage system" in the brain, which is thought to be responsible for maintaining GPX expression. Further evidence of the role of the SeP-GPX axis and ApoER2 in ferroptosis and chemotherapy resistance are needed.

Drugs that inhibit SeP expression have yet to be identified. We recently found that sulforaphane and epigallocatechin gallate suppress SeP expression in hepatocytes. SeP expression is known to contribute to the risk of colorectal cancer^54^, prostate cancer^55^, and pancreatic cancer^56^, and may show an important role not only in GBM but also in other cancers. We would like to find an inhibitor of SeP in GBM by performing drug screening and verifying whether it leads to improvement of GBM resistance to treatment.

## Materials and methods

### Chemicals

RSL3 was obtained from Selleck (Houston, USA), selenite was from Nacalai tesq (Kyoto, Japan), and RPMI 1640 medium was purchased from Merck (Darmstadt, Germany). Dulbecco’s modified Eagle’s medium (DMEM), penicillin and streptomycin, and fetal bovine serum (FBS) were obtained from Invitrogen (Thermo Fisher Scientific, MA, USA). All other chemicals used were of the highest quality commercially available.

### Data analysis for RNA sequence of GBM patients

Fastq files from GSE86202 were utilized for this study^57^. Short reads were aligned to the human genome (hg38) using HISAT2. Transcript per million (TPM) values were calculated using DESeq2. GRCh38 was used as the reference genome and mapped using STAR. Results were normalized with DESeq2 to obtain expression comparison data. These operations and visualizations were performed with R version 4.2.2 and Python.

### Cell culture and transfection

T98G, YKG1 and A172 cells (obtained from Institute of Development, Aging and Cancer, Tohoku University), or U87 and U251 cells (obtained from JCRB cell bank) were cultured with RPMI 1640 medium supplemented with 10% fetal bovine serum and 1% penicillin streptomycin in 5% CO_2_, at 37 °C. Patient-derived cell studies were approved by the ethics committee of the faculty of medicine at Tohoku University (Approved No. 2023-1-321). The patient tissue from which the cells were donated was confirmed to have MGMT promoter methylation and null p53 expression from pathological diagnosis. We obtained informed consent from the patient for the culture of patient-derived primary cells. The tumor cells were isolated from a GBM patient at Tohoku university hospital and cultured using EMEM supplemented with 10% fetal bovine serum and 1% penicillin–streptomycin. Gene-specific and negative control siRNAs were purchased from Sigma (MO, USA). Lipofectamine™ RNAiMAX Transfection Reagent (Thermo Scientific, MA, USA) was used to transfect siRNA into cells (10 nM siRNA and 1 μl Lipofectamine/ml), and the cells were harvested for subsequent treatments after 48 h of transfection. The target sequence of the SeP gene by siRNA is as follows. SeP#1 5′-GCAUAUUCCUGUUUAUCAA-3′. SeP#2 5′-GCAUAUUCCUGUUUAUCAA-3′. SeP#3 5′-GCAUACUGCAGGCAUCUAA-3′.

### RNA extraction and quantitative PCR

After treatment, the cell culture medium was discarded, cells were washed with PBS, and ISOGEN II (NIPPON GENE, Tokyo, Japan), an RNA extraction reagent, was added. RNA was purified following the manufacturer’s instructions, concentration was determined by NanoDrop (Thermo), and reverse transcription was performed using a PrimeScript RT Reagen Kit (Takara Bio Inc, Shiga, Japan). The reagent Power SYBR™ Green PCR Master Mix (Thermo Fisher Scientific, USA) and a thermal cycler (CFX Connect™, Bio-Rad, CA, USA) were used for quantitative PCR (qPCR). All data are normalized by each of *GAPDH* mRNA level.

The primers used in this study are as follows.

*GAPDH*: F-GCACCGTCAAGGCTGAGAAC, R-TGGTGAAGACGCCAGTGGA

*SELENOP*: F-CCCCCAGCCTGGAGCATAAG, R-TGCACAGGTATCAGCTGGCTT

*GPX1*: F-CAGTCGGTGTATGCCTTCTCG, R-GAGGGACGCCACATTCTCG

*GPX4*: F-GAGGCAAGACCGAAGTAAACTAC, R-CCGAACTGGTTACACGGGAA

### Cell viability

After transfection of SeP or negative control siRNA for 24 h, the culture medium was replaced and treated with RLS3 and selenite for an additional 24 h. After that, medium were changed with 10% alamarBlue™ Cell Viability Reagent (Thermo Fisher Scientific), 90% complete DMEM and further incubated for 2 h. After the incubation, a microplate reader (SpectraMax iD5, Molecular Device, MA, USA) was used to detect the fluorescence intensity at Ex 544/Em 585 nm. The data are shown as a ratio, with the control as 1.

### Western blotting

Cellular proteins were extracted with RIPA lysis buffer (50 mmol/L Tris–HCl Buffer pH 7.6, 150 mmol/L NaCl, 1% Nonidet P40 Substitute, 0.5% Sodium Deoxycholate, 0.1% SDS). Protein concentration was determined by DC protein assay reagents according to the manufacturer’s instructions (Bio-Rad). Then, sample loading buffer was added and an aliquot of proteins were denatured at 95 °C for 10 min, separated by SDS polyacrylamide gel electrophoresis, and transferred to PVDF membranes. Blocking was performed with 5% skim milk for 1 h. The membrane was rinsed with TBST and cut around the target protein molecular weight to suppress antibody volume, then incubated with indicated primary antibodies (anti-GPX1 ab22604, and anti-GPX4 ab125066 were from Abcam, Cambridge, UK; anti-GAPDH 015-25473 was from Wako pure chemical, Osaka, Japan). After washing with TBST, the membrane was incubated with a secondary antibody (DAKO, Nowy Sącz, Poland) for 1 h at room temperature. Finally, protein bands were detected using an ImmunoStar LD kit (FUJIFILM Wako Pure Chemical Corporation, Japan) and Luminograph (ATTO, Tokyo, Japan). Full images of WB were shown in supplemental information (Figs. S5–S7).

### Statistical analysis

Each experiment was repeated at least three times independently, and the experimental data were expressed in the form of mean ± standard deviation. GraphPad Prism 9 was used to analyze the experimental results. The comparison between two groups was carried out by t-test, and the comparison between multiple groups was carried out by Dunnett’s test or Tukey’s HSD analysis. P ≤ 0.05 means it is statistically significant.

### Ethical approval

All experiments were performed in accordance with the relevant guidelines and regulations. All human experiments were approved by the ethics committee of Tohoku University.

### Consent to participate

All authors checked the study and agreed to participate in the manuscript.

### Supplementary Information

Supplementary Figures.

## Data Availability

All data needed to evaluate the conclusions in the paper are present in the paper or in the Supplementary Materials.
