# Supplementary material for: Selenoprotein P expression in glioblastoma as a regulator of ferroptosis sensitivity: preservation of GPX4 via the cycling-selenium storage
Source: Sci Rep. 2024 Jan 5;14:682. doi: 10.1038/s41598-024-51259-5 (PMC10770386; doi:10.1038/s41598-024-51259-5)

Supplemental Information

Selenoprotein P expression in glioblastoma as a regulator of ferroptosis sensitivity: preservation of GPX4 via the cycling-selenium storage

Quantitative PCR

RNA extraction and reverse transcription was performed as same as in the main manuscript. The following primers were attritionary used for supplemental data Fig. S2.

MDM2: F-GAATCATCGGACTCAGGTACATC, R-TCTGTCTCACTAATTGCTCTCCT

MDM4: F-CAGCACATGACGGAGGTTGT, R-TCATCCAAATACTCCACACGC

TP53: F-TGATTGTCGAAGAACCATTTCGG, R-TGCAGGGATCAAAAAGTTTGGAG

Supplemental Fig S1.

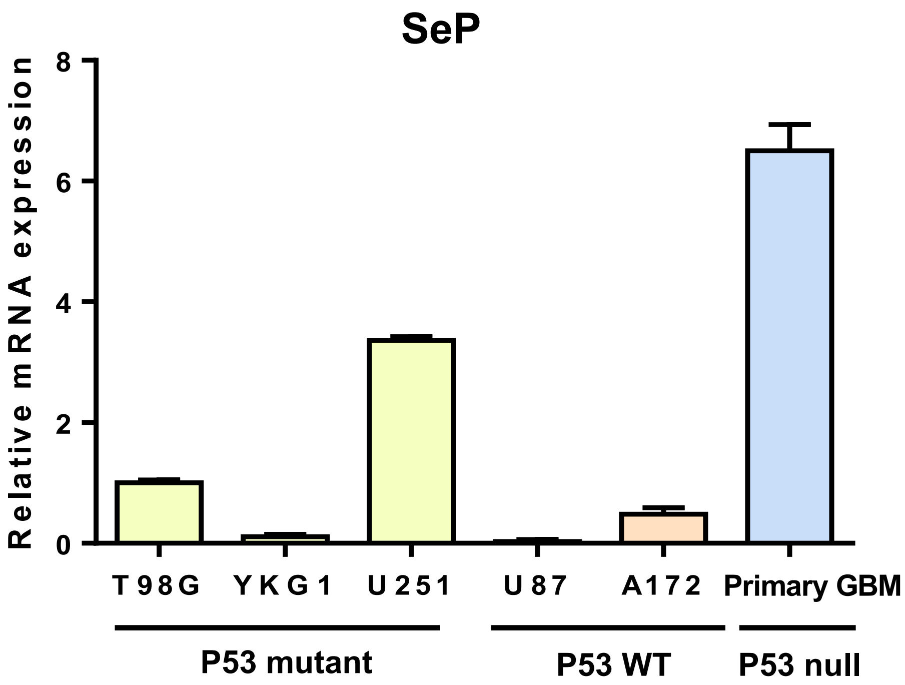

Figure S1, SeP expression in various GBM cell line.

The cells were cultured and extracted mRNA were subjected to qPCR analysis. mRNA levels of SeP were shown as mean±S.D.; n=3, and the relative value as control as 1.

Supplemental Fig S2.

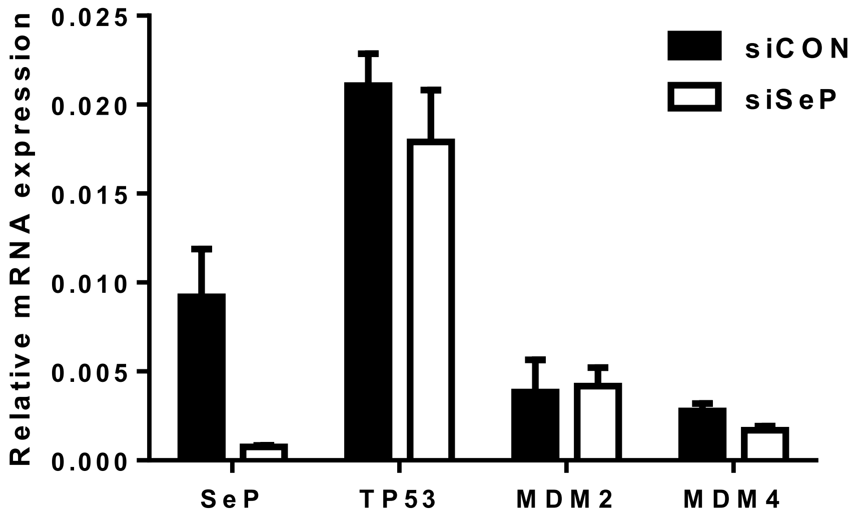

Figure S2, Effect of SeP on the regulation of TP53. After knockdown of SeP of T98G cell, mRNA expression of TP53, MDM2,MDM4 genes was detected by qPCR. The data were shown as mean ± S.D.; n=3, and the relative value as control as 1.

Supplemental Fig S3.

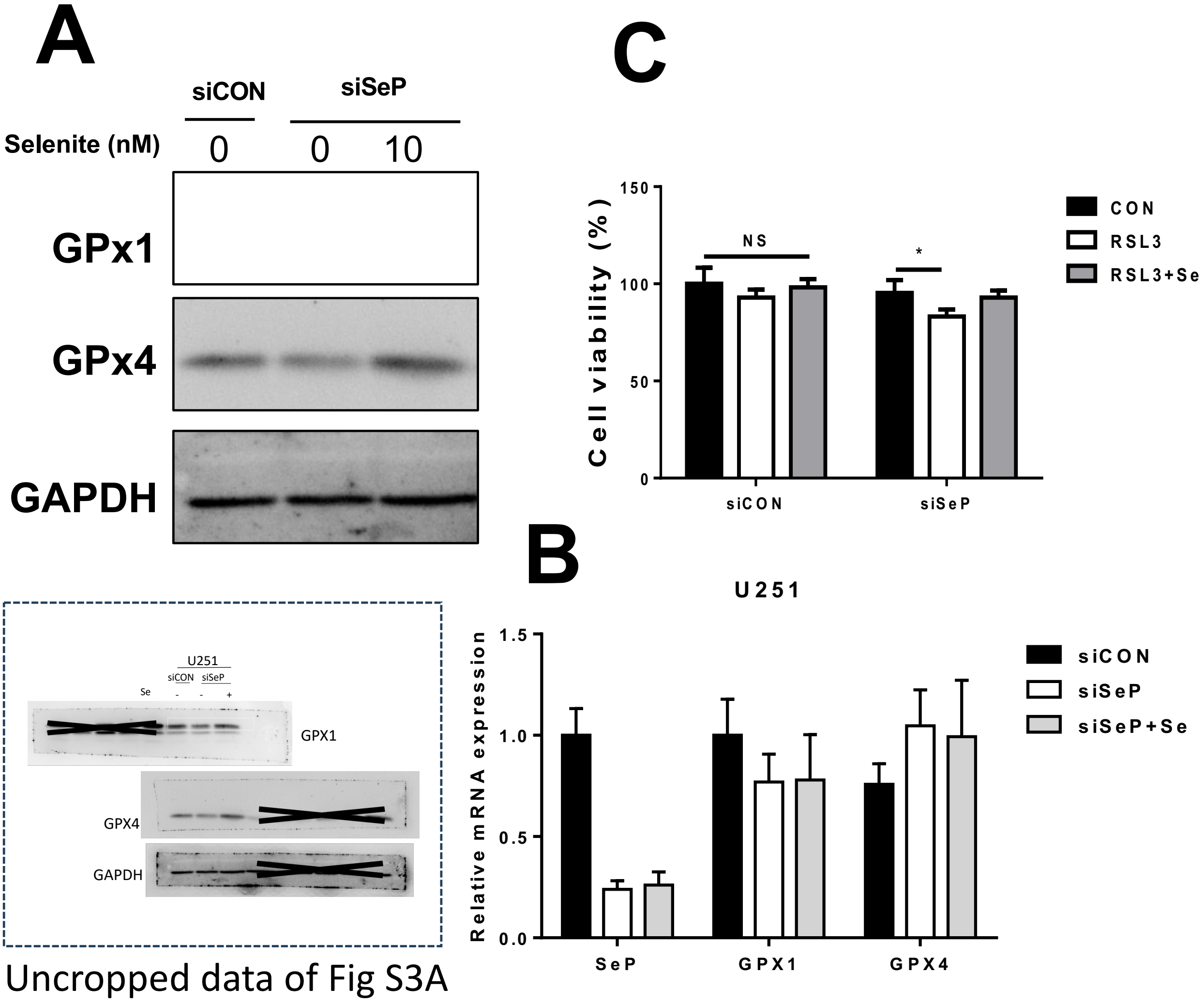

Figure S3. **Effect of SeP on selenium utilization and ferroptosis resistance of U251 cells.** U251 cells were transfected with siRNA for 24 hours and incubated with the indicated concentration of selenite for an additional 24 hours. The protein was extracted, and GPx protein levels were detected by Western blot (A) and mRNA level was detected by qPCR (B). After SeP was knocked down, RSL3 or selenite 10 nM was added and incubated for 24 hours before cell viability was measured (C). Data are presented as the mean ± S.D. ; n=3. Statistical significance was assessed by Tukey's HSD(C). **p* < 0.05 vs control.

Supplemental Fig S4.

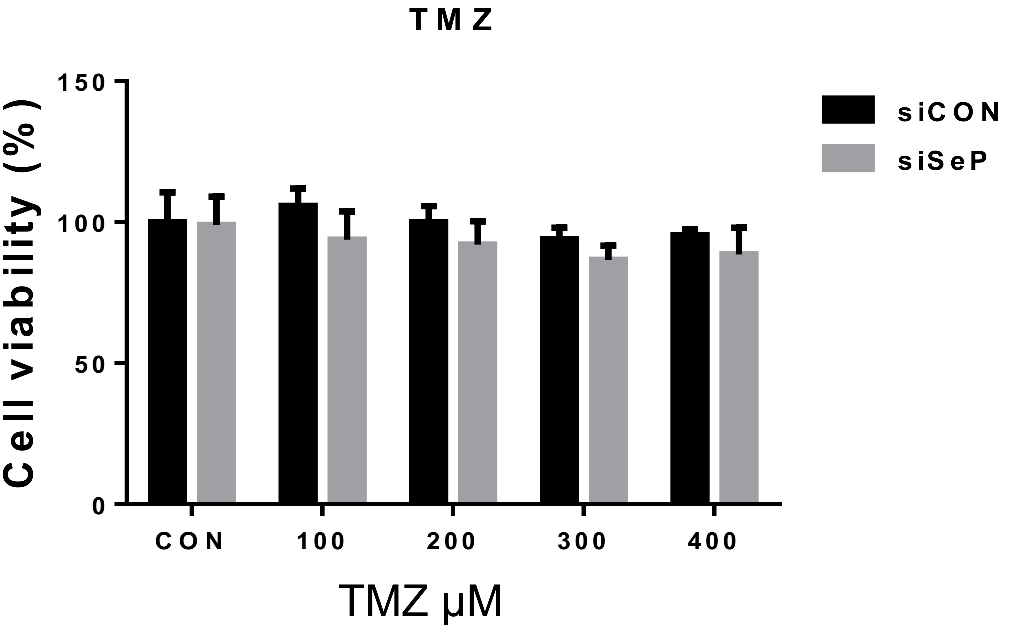

Figure S4. **Effect of SeP on TMZ resistance of T98G cells.** T98G cells were transfected with siRNA for 24 hours and TMZ was added and incubated for 24 hours before cell viability was measured by alamarBlue assay. Data are presented as the mean ± S.D. (n=3).

Supplemental Fig S5.

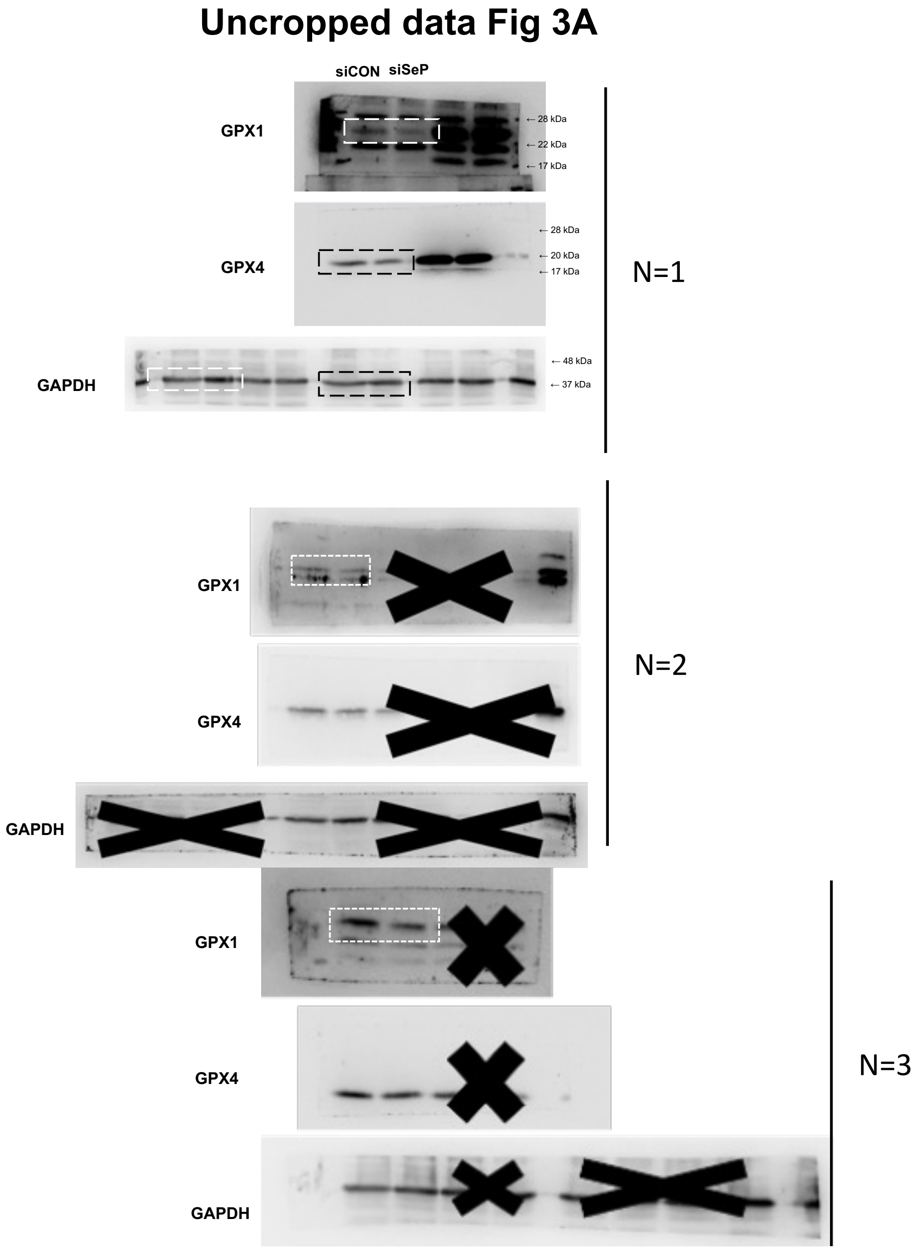

Supplemental Fig S6.

Uncropped data Fig 4A

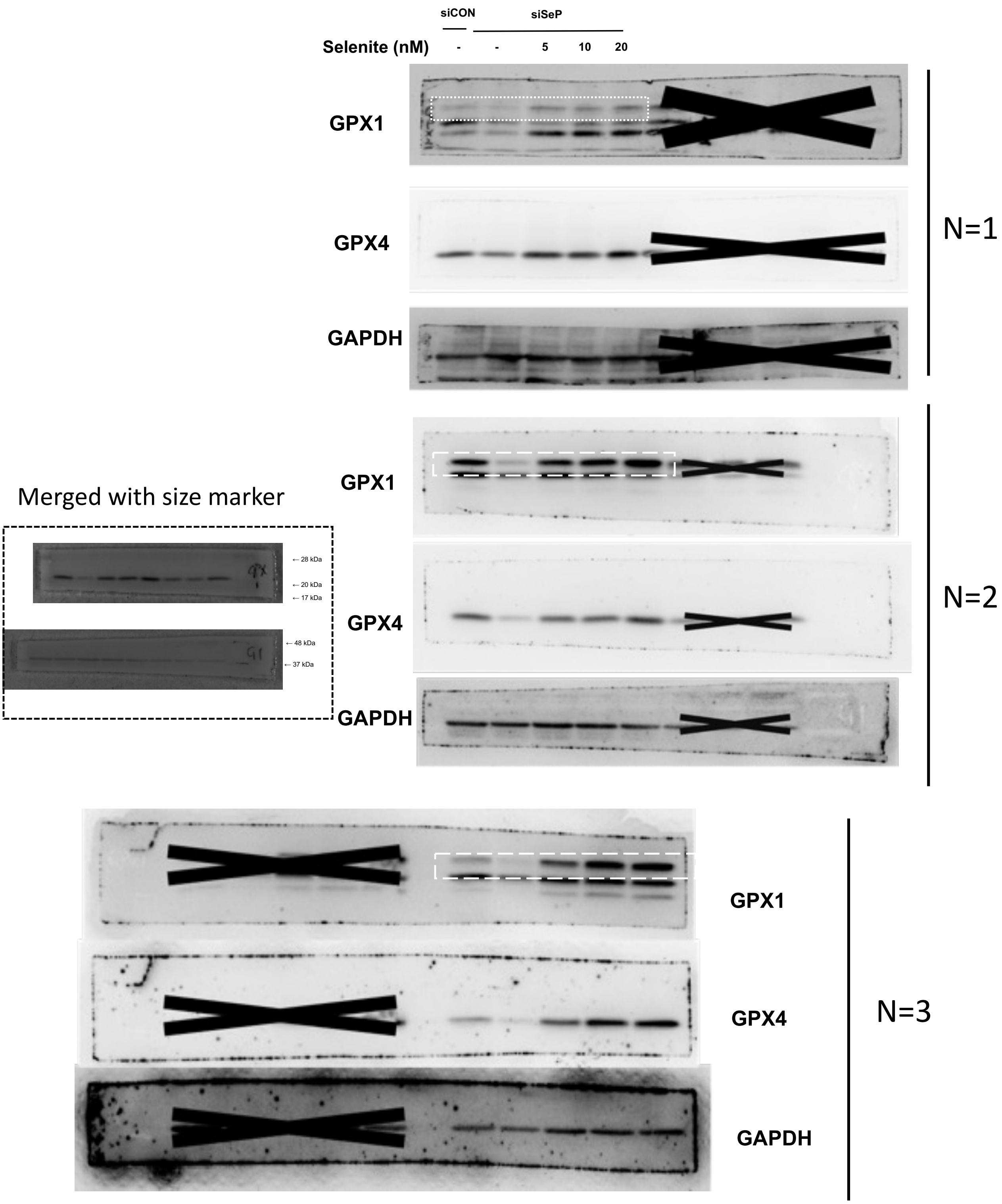

Supplemental Fig S7.

Uncropped data Fig 5E

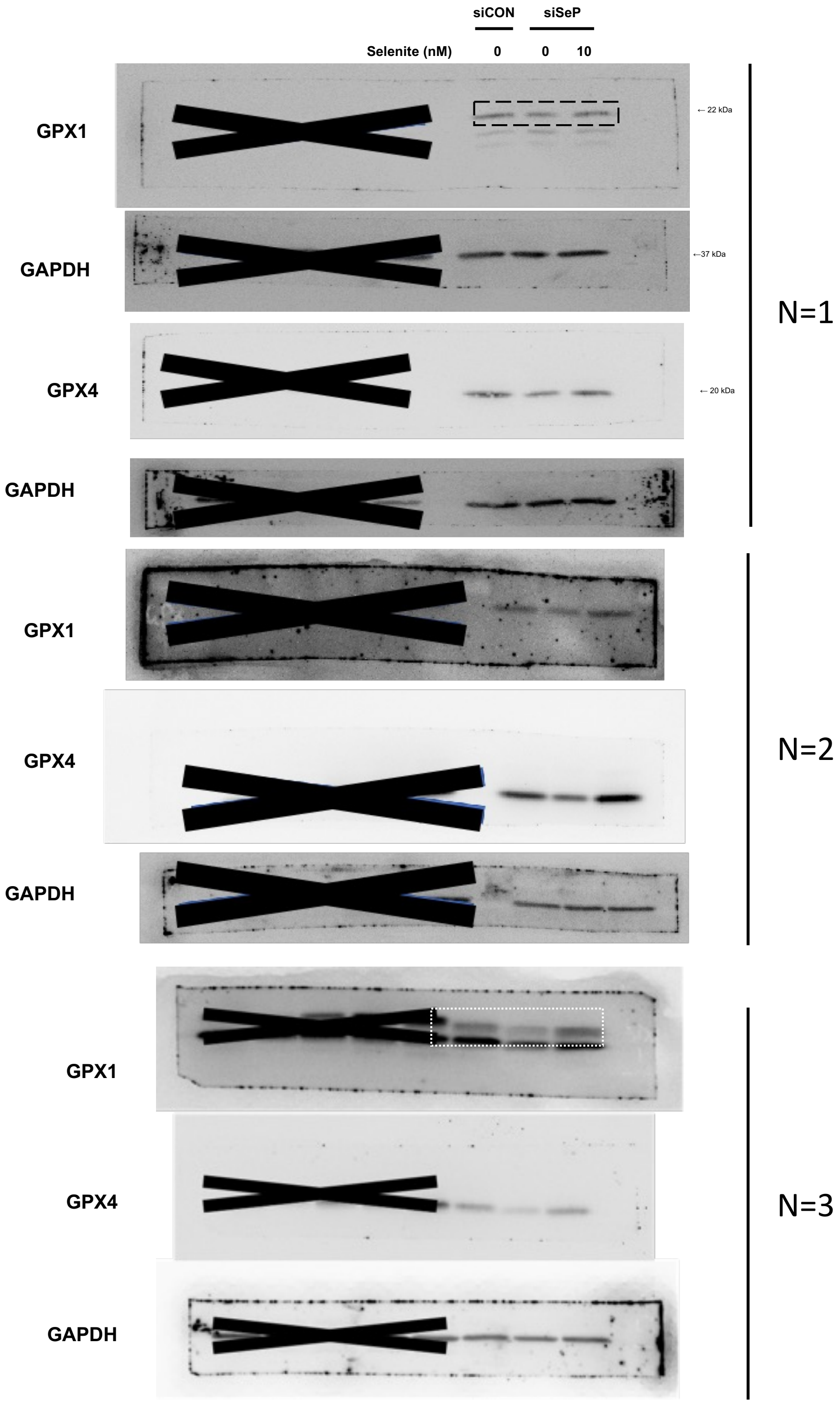

Supplement: Supplementary file 1 — Supplementary Figures. [file 41598_2024_51259_MOESM1_ESM.pdf]
